# LncRNA SOX2OT alleviates mesangial cell proliferation and fibrosis in diabetic nephropathy via Akt/mTOR-mediated autophagy

**DOI:** 10.1186/s10020-021-00310-6

**Published:** 2021-07-08

**Authors:** Ke Chen, Bo Yu, Jie Liao

**Affiliations:** 1 Department of Geriatrics, Xiangya Hospital, Central South University, Changsha, 410008 Hunan People’s Republic of China; 2 National Clinical Research Center for Geriatric Disorders, Xiangya Hospital, Central South University, Changsha, Hunan 410008 People’s Republic of China

**Keywords:** Akt, Autophagy, Diabetic nephropathy, MTOR, SOX2OT

## Abstract

**Background:**

Accumulating evidences have demonstrated that long non-coding RNAs (lncRNAs) are involved in the pathophysiology of diabetic nephropathy (DN). lncRNA SOX2OT plays an essential role in many diseases, including diabetes. Herein, we aim to investigate the underlying mechanism of lncRNA SOX2OT in DN pathogenesis.

**Methods:**

Streptozotocin-induced DN mouse models and high glucose-induced mouse mesangial cells were constructed to examine the expression pattern of lncRNA SOX2OT. The activation of autophagy was evaluated using immunohistochemistry, immunofluorescence and western blot analysis, respectively. SOX2OT overexpressing plasmid was applied to further verify the functional role of SOX2OT in DN pathogenesis. CCK-8 and EDU assays were performed to the proliferation of mesangial cells. Additionally, rapamycin, the inhibitor of mTOR signaling, was used to further clarify whether SOX2OT controls DN development through Akt/mTOR pathway.

**Results:**

lncRNA SOX2OT was markedly down-regulated both in streptozotocin-induced DN mice and high glucose-induced mouse mesangial cells. Moreover, overexpression of lncRNA SOX2OT was able to diminish the suppression of autophagy and alleviate DN-induced renal injury. Functionally, CCK-8 and EDU assays indicated that lncRNA SOX2OT overexpression significantly suppressed the proliferation and fibrosis of mesangial cells. Additionally, an obvious inhibition of Akt/mTOR was also observed with lncRNA SOX2OT overexpression, which was then further verified in vivo.

**Conclusion:**

In summary, we demonstrated that lncRNA SOX2OT alleviates the pathogenesis of DN via regulating Akt/mTOR-mediated autophagy, which may provide a novel target for DN therapy.

**Supplementary Information:**

The online version contains supplementary material available at 10.1186/s10020-021-00310-6.

## Introduction

Diabetic nephropathy (DN) is one of the most serious microvascular complications induced by diabetes, which is a key cause of kidney failure (Tan et al. 2019). The primary pathological manifestations of DN include glomerular hypertrophy, basement membrane thickening, and increased extracellular matrix, which are caused by the proliferation of mesangial cells and changes in the renal microenvironment (Wang et al. 2018; Nagai et al. 2005). Ultimately, DN leads to renal fibrosis and chronic renal failure (Wang et al. 2018; Yang et al. 2019). Glomerular mesangial cells are the basic components of the glomerulus. They are the main cells responsible for the synthesis of matrix proteins, which can lead to glomerular hypertrophy (Han et al. 2017; Huang et al. 2019). Glomerular morphological changes are one of the pathological damages of DN. Therefore, inhibiting the proliferation of mesangial cells is considered an effective strategy for optimizing the diagnosis and treatment of DN (Wang et al. 2016). However, the molecular mechanisms that cause these changes in the microenvironment remain largely unknown. Investigating the underlying mechanism of DN is of great significance for the development of more effective diagnostic and treatment strategies.

Autophagy, also referred to as type II programmed cell death, is an important process involving lysosomal-mediated degradation of intracellular components (Ouyang et al. 2012). The Akt/mTOR signalling pathway is a crucial regulator of autophagy (Yu et al. 2010; Sui et al. 2017; Kim et al. 2017), which also plays an important role in tumourigenesis (Fruman and Rommel 2014). Autophagy is reportedly a vital factor in related complications of diabetes (Deshpande et al. 2018) (Lim et al. 2018). For example, Deshpande et al. observed that a microRNA-mediated signalling cascade reduced autophagy in diabetes-induced renal glomerular hypertrophy (Deshpande et al. 2018). However, effective treatments for autophagy-related diabetes are currently lacking.

Long non-coding RNAs (lncRNAs) are non-coding RNAs with a length of more than 200 nucleotides (Long and Danesh 2018). Current studies have shown that lncRNAs play biological roles as potential therapeutic targets in various diseases, including DN (Wang et al. 2016) (Wang et al. 2016a, b; Gao et al. 2018). Lnc RNA SOX2 overlapping transcript (SOX2OT), a lncRNA transcribed in the same orientation as SOX2, is mapped to human chromosome 3q26.3 (Chr3q26.3) locus (Shahryari et al. 2015). SOX2OT is highly conserved between mouse and human (Fantes et al. 2003). Recent studies identified that SOX2OT expression is increased in many cancers (Han et al. 2018; Zhang et al. 2016). A recent microarray analysis indicated that SOX2OT is downregulated in DN (Zhang et al. 2018), and this result was further confirmed at the cellular level. Zhang et al. reported that SOX2OT alleviated high glucose-induced podocytes injury via autophagy induction by miR-9/SIRT1 axis (Zhang et al. 2019). However, the specific molecular mechanisms mediated by SOX2OT in the pathogenesis of DN require further exploration.

Our current study demonstrated that SOX2OT expression is reduced in both DN mice and mouse mesangial cells treated with high glucose, and its overexpression dramatically promoted autophagy and reduced kidney damage. More importantly, we discovered for the first time that SOX2OT regulates autophagy through the Akt/mTOR pathway in glomerular mesangial cells. Hence, we propose that SOX2OT alleviates cell proliferation and fibrosis in DN by regulating Akt/mTOR-mediated autophagy, indicating that SOX2OT may represent a novel target for DN treatment.

## Materials and methods

### Animal model

The DN animal model was constructed as described by Huang et al. and Wang et al. (Huang et al. 2019; Wang et al. 2019). Specific-pathogen-free (SPF) grade male C57BL/6 mice (aged 6–8 weeks old, 20–24 g), obtained from Slac-Jingda Laboratory Animal (Hunan, China), were maintained in an SPF lab at 22–23 °C, 55–60% humidity and a 12 h light (7:00)/dark (19:00) cycle. Mice were weighted and the fasting blood glucose for 12 h was measured. Streptozotocin (STZ) (Sigma Aldrich, St. Louis, MO, USA) was dissolved in 0.1 M citrate acid buffer (pH 4.0, Sigma, St. Louis, MO). DN mice were created by injecting 100 mg/kg STZ once per day. Three days later, the blood glucose was measured and mice with a blood glucose more than 16 mmol/L were considered successful DN models. Two weeks later, DN mouse model was confirmed again by measuring blood glucose and four groups were established (n = 10/group): control, DN, DN + pcDNA-vector, and DN + pcDNA-SOX2OT. Mice received an intravenous injection of 100 μl lentivirus (1 × 10^8^ TU/mL) carrying pcDNA-vector or pcDNA-SOX2OT. The intraperitoneal injection of rapamycin (MCE, HY-10219) was given two weeks later (2 mg/kg/day, daily). After 4 weeks, all mice were placed in metabolic cages for urine collection to measure 24 h proteinuria. The mice were then weighted and anesthetized with 30 mg/kg of sodium pentobarbital (Sigma) and sacrificed by decollation. The blood was collected from the abdominal aorta and the serum was separated from the cells by centrifuging the blood at 3000×*g* at 20 °C for 15 min. Blood glucose was measured with blood drawn from the tail vein using a glucose analyzer (Roche, Germany). Serum creatinine and blood urea nitrogen were determined by an automatic biochemistry analyzer (CD-1600CS, Abbott Labs, USA). The kidneys were taken out immediately, and the left kidney was weighed. One kidney was placed in liquid nitrogen and stored at − 80 °C, and the other kidney was fixed with 4% paraformaldehyde. All animal experiments were performed according to the Guide for the Care and Use of Laboratory Animals of the Chinese National Institutes of Health. The current study was approved by the Animal Ethics Committee of Xiangya Hospital of Central South University, Changsha.

### Cell culture

Mouse mesangial cells were purchased from American Type Culture Collection (ATCC, Manassas, VA). Cells were cultured in Dulbecco’s modified Eagle’s medium with 10% foetal bovine serum (Invitrogen, CA), penicillin (100 U/ml), and streptomycin (100 μg/ml) in a humidified 5% CO_2_ atmosphere.

### Transfection

SOX2OT sequences were synthesized by GenePharma (Shanghai, China). To overexpress SOX2OT, the SOX2OT cDNA fragments were cloned into the pcDNA 3.1 vector (GenePharma, Shanghai, China). The empty pcDNA3.1 vector was used as control. Transfection of pcDNA-SOX2OT or pcDNA-vector into cells was performed using Lipofectamine 2000 (Invitrogen, Carlsbad, CA, USA) according to the manufacturer’s protocol. The constructed vectors and and the lentiviral vector pMD2.G were digested with BamHI/EcoRI. Ligation was performed to construct SOX2OT overexpression lentiviral recombinant plasmid. The lentivirus packaging was performed by Auragene Biotechnology Company (Changsha, China).

### Haematoxylin and eosin (HE) staining

Renal tissues were fixed in 10% paraformaldehyde (Sigma-Aldrich; Merck KGaA) for 48 h. Tissues were then washed with distilled water for 1 h, dehydrated in an ascending series of ethanol, embedded in paraffin and gently sliced into 5-μm-thick sections. Renal sections were routinely dewaxed in xylene and rehydrated in a descending series of alcohol. Next, samples were stained with haematoxylin for 5 min followed by five dips in 1% acid ethanol. After rinsing in distilled water, samples were stained with eosin for 3 min. Finally, renal tissues were dehydrated with graded ethanol and cleared in xylene. Images were obtained using ImageJ software under an optical microscope.

### Masson staining

Renal sections were stained with a Masson assay kit (Shanghai Yuanye Biotechnology Co., Shanghai, China) according to the manufacturer's instructions. Samples were then observed under an optical microscope. Areas stained in blue indicate collagen fibres, while areas stained red indicate cell matrix.

### Immunohistochemistry (IHC)

Kidney tissue sections were deparaffinized and incubated in peroxidase solution (3% H_2_O_2_) followed by incubation at room temperature for 30 min with non-immune animal serum. Next, slides were incubated with anti-LC3 antibody (1:200, Abcam), anti-fibronectin antibody (1:500, Abcam) or anti-collagen IV antibody (1:500, Abcam) at 4 °C overnight. Slides were then incubated with a second antibody conjugated to horseradish peroxidase (HRP) for 1 h and washed with phosphate-buffered saline.

### Cell counting kit-8 (CCK-8) assay

CCK-8 assay was performed to assess cell viability according to the manufacturer’s instructions (Dojindo Molecular Technologies, Dojindo, Japan). Briefly, cells were seeded into 96-well plates at a density of 3000 cells/well. At different time points after transfection, 100 μl CCK-8 solution was added into each well for 2 h at 37 °C. Absorbance was measured at 450 nm in a microplate reader (Molecular Devices).

### 5-Ethynyl-2′-deoxyuridine (EDU) assay

EDU assay was used to examine cell proliferation (RiboBio, Nanjing, China). Briefly, cells were transfected for 48 h and then treated with 50 μM EDU. Apollo staining and DAPI staining were performed to observe EDU-positive cells.

### Immunofluorescence

Cells incubated in 24-well plates were washed with PBS and fixed with 4% formaldehyde solution for 30 min (Sigma). Cells were permeabilized with 0.2% Triton X-100 for 5 min. After blocking in 5% BSA (Sigma) for 1 h, samples were incubated with primary antibody against LC3 (1:100, Abcam) at 4 °C overnight and subsequently incubated with fluorescence-conjugated secondary antibody for 1 h. Cells were then incubated with DAPI for 5 min at room temperature and were subsequently detected under a fluorescence microscope (Leica, Germany).

### Total RNA extraction, reverse transcription and real-time quantitative PCR

Total RNA was extracted from mesangial cells and kidney cortical tissues using TRIzol reagent (Invitrogen), and cDNA was synthesized by a Mir-X^TM^miRNA First Strand Synthesis Kit (Takara Bio Technology, Dalian, China). DBI SYBR Premix Ex Kit (TaKaRa Bio Technology, Dalian, China) was used for quantitative PCR analysis. Primer sequences were as follows: SOX2OT forward, 5ʹ-GCTCGTGGCTTAGGAGATTG-3ʹ; SOX2OT reverse, 5ʹ-CTGGCAAAGCATGAGGAACT-3ʹ; GAPDH forward, 5ʹ-ACCACAGTCCATGCCATCAC-3ʹ; GAPDH reverse, 5ʹ-TCCACCACCCTGTTGCTGTA-3ʹ. GAPDH was detected as an internal control. The 2^−ΔΔCt^ method was used for quantitative PCR analysis.

### Western blot

RIPA lysis buffer was used to extract total protein from mesangial cells and homogenates of the cortical tissues. Protein concentration was determined using a BCA protein assay (Thermo Fisher Scientific). Equal amounts of protein (40 μg/lane) were separated by 10% SDS-PAGE gels and transferred to PVDF membrane (Millipore, USA). Membranes were blocked in 5% non-fat milk for 1 h. Membranes were then incubated with the following antibodies at 4 °C overnight: anti-LC3I and anti-LC3II (1:1000, Abcam), anti-Beclin1 (1:1000, Abcam), anti-p62 (1:1,000, Abcam), anti-TGF beta-1 (1:1000, Abcam), anti-fibronectin (1:1000, Abcam), anti-ASK1 (1:1000, Abcam), anti-collagen IV (1:1000, Abcam), anti-α-SMA (1:400, Abcam), anti-E-cadherin (1:1000, Abcam), anti-N-cadherin (1:1000, Abcam), anti-p-Akt (1:1000, Abcam), anti-p-mTOR (1:1000, Abcam), anti-Akt (1:1000, Abcam), anti-mTOR (1:1000, Abcam), and anti-GAPDH (1:5000, Abcam, Cambridge, UK). Membranes were washed with TBST (Beyotime Biotechnology, Shanghai, China) and incubated with horseradish peroxidase (HRP)-labelled secondary antibodies (1:20,000, BOSTER, Beijing, China) at room temperature for 45 min. Target signals were observed with enhanced chemiluminescence (ECL) detection (Pharmacia; Piscataway-USA).

### Statistical analysis

All data in this study are presented as the mean ± standard deviation (SD) of three independent experiments. GraphPad Prism 6 (La Jolla, CA, USA) was used for statistical analysis. Student's t-test (two-tailed) was performed when two groups were compared. One-way-ANOVA followed by Newman-Keuls post hoc test was performed for multi-group comparison. P-values < 0.05 were considered statistically significance.

## Results

### Reduced SOX2OT and decreased autophagy were observed in STZ-induced DN mice

**Fig. 1:**
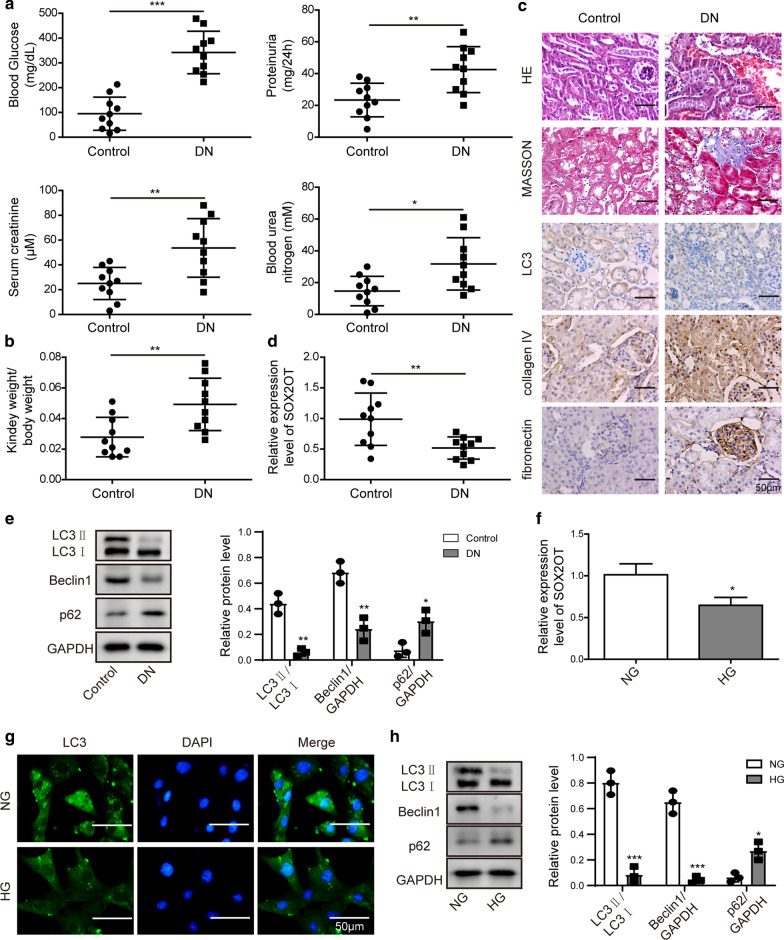
SOX2OT expression and autophagy in STZ-induced DN mice. **a** Blood glucose, 24 h proteinuria, serum creatinine and blood urea nitrogen levels in streptozotocin (STZ)-induced DN mice and normal mice. **b** Relative kidney weight in DN mice compared to the control group. **c** HE, Masson and LC3 staining of renal sections. IHC staining detected fibronectin and collagen IV levels. Scale bar: 50 μm. **d** RT-qPCR analysis of SOX2OT mRNA expression in the kidney of DN mice compared to the control group. **e** Western blot analysis of LC3II/LC3I, Beclin1 and p62 in the kidney of DN mice compared to the control group. **f** SOX2OT mRNA expression in mouse mesangial cells detected by RT-qPCR. Cells were incubated in high glucose (HG, 30 mM) or normal glucose (NG, 5.5 mM). **g** Immunofluorescence analysis of LC3 in mouse mesangial cells treated with NG or HG. Scale bar: 50 μm. **h** Protein levels of LC3II/LC3I, Beclin1 and p62 in mouse mesangial cells treated with NG or HG were determined by western blot. GAPDH was used as a loading control in western blot analysis. Error bars represent the mean ± SD from three independent experiments. *p < 0.05, **p < 0.01, ***p < 0.001, Student's t-test (two-tailed). HG, high glucose. NG, normal glucose

To determine the effect of SOX2OT on DN, we first measured blood glucose, 24 h proteinuria, serum creatinine and blood urea nitrogen in streptozotocin (STZ)-induced DN mouse models. As shown in Fig. 1a, these indicators were markedly increased in DN mice compared to that of control, reflecting damage to the kidneys. We also found that, compared to the control group, relative kidney weight in DN mice was dramatically increased (Fig. 1b). Renal pathological changes were then detected by HE and Masson staining. In the control group, the kidney tissue appeared as a clear tubular structure with normal glomeruli. The renal tubular epithelial cells were neatly arranged, and the basement membranes were complete. There was no interstitial inflammatory cell infiltration observed in normal kidney tissues (Fig. 1c). However, enlarged glomeruli were observed in DN mice and the lumen of the renal tubules was dilated by an irregularly thickened basement membrane. There was obvious vacuolarization of renal tubular epithelial cells and interstitial inflammatory cell infiltration. An increase in areas that were strongly positive for Masson staining was also observed, indicating apparent interstitial fibrosis. Immunohistochemical staining further verified that LC3 level was notably inhibited, while the expression of fibronectin and collagen IV was significantly up-regulated in the DN group (Fig. 1c). Moreover, RT-qPCR assay implied that SOX2OT was decreased in the kidney tissues of DN group (Fig. 1d). Next, autophagy-related molecules were measured by western blot analysis. Results demonstrated that, compared with the control group, the ratio of LC3II/LC3I and levels of Beclin1 in the kidney of DN mice were dramatically suppressed, while expression of p62 was notably upregulated (Fig. 1e). Furthermore, mouse mesangial cells were treated with high glucose (HG, 30 mM) or normal glucose (NG, 5.5 mM), and RT-qPCR results revealed that SOX2OT was greatly reduced in HG cells as well (Fig. 1f). In addition, immunofluorescence assay indicated that HG treatment significantly reduced LC3 fluorescence intensity (Fig. 1g). Western blot analysis indicated downregulation of LC3II/LC3I and Beclin1 but upregulation of p62 in HG cells compared to NG cells (Fig. 1h). Collectively, these data suggest that SOX2OT may play a critical role in DN.

### SOX2OT overexpression promoted activation of autophagy and relieved DN-induced renal injury

**Fig. 2:**
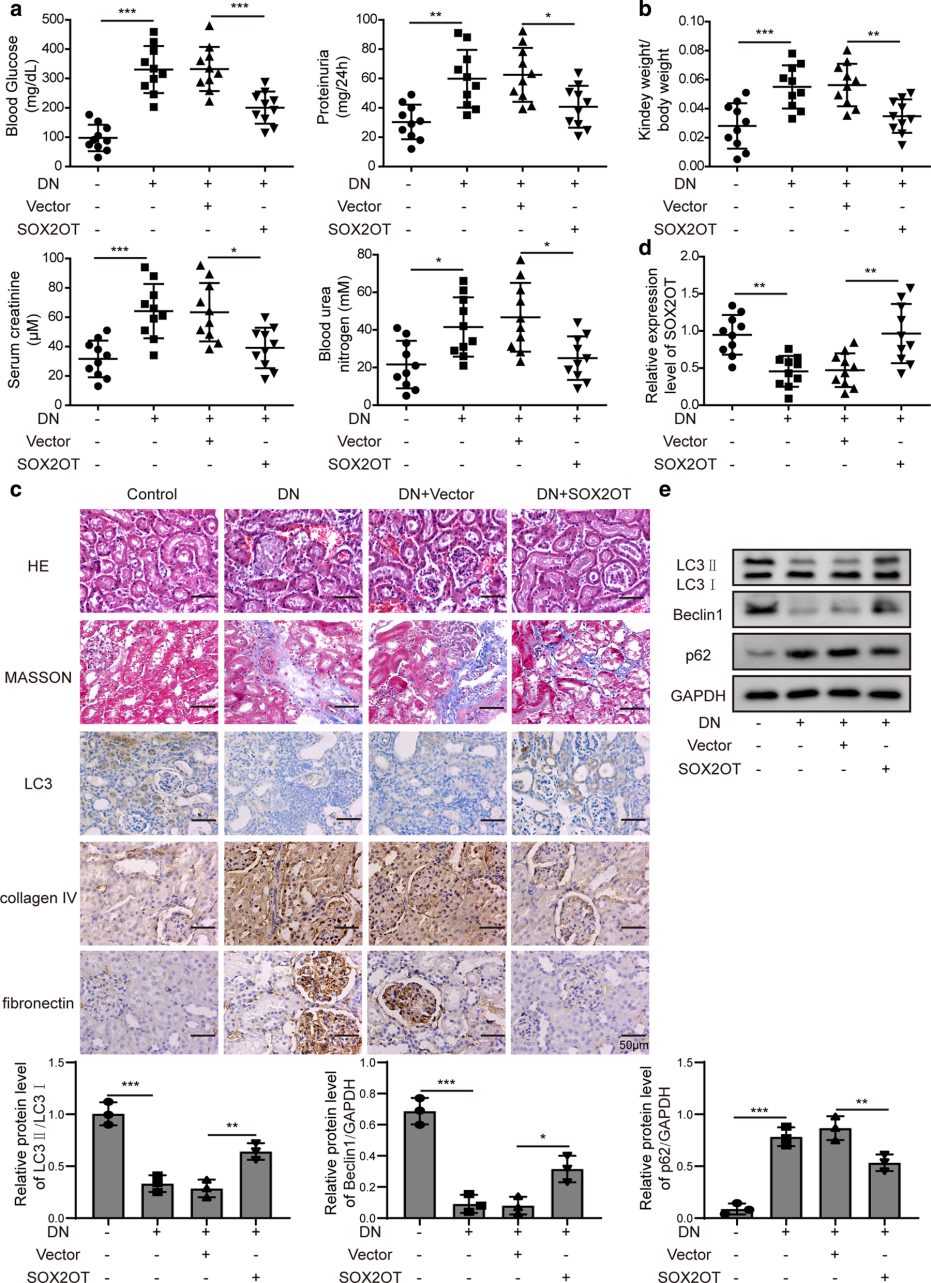
SOX2OT overexpression promoted autophagy and relieved DN-induced renal injury in vivo. DN mice were injected with lentivirus carrying pcDNA-SOX2OT or pcDNA-vector. **a** Blood glucose, 24 h proteinuria, serum creatinine, blood urea nitrogen levels in DN mice after SOX2OT overexpression. **b** Relative kidney weight in DN mice in response to SOX2OT overexpression. **c** HE, Masson and LC3 staining of renal sections in DN mice with SOX2OT overexpression. IHC staining detected fibronectin and collagen IV levels. Scale bar: 50 μm. **d** SOX2OT mRNA expression was determined by RT-qPCR in the kidney of DN mice with SOX2OT overexpression. **e** Western blot analysis of LC3II/LC3I, Beclin1 and p62 in the kidney of DN mice after SOX2OT overexpression. GAPDH was used as a loading control in western blot analysis. Error bars represent the mean ± SD from three independent experiments. *p < 0.05, **p < 0.01, ***p < 0.001, One-way-ANOVA followed by Newman-Keuls post hoc test

To explore the function of SOX2OT in DN, we overexpressed SOX2OT in vivo by injection with lentivirus carrying SOX2OT plasmid (pcDNA-SOX2OT) into DN mice. We then examined blood glucose, 24 h proteinuria, serum creatinine, blood urea nitrogen and relative kidney weight. As shown in Fig. 2a, b, these indicators were much higher in DN mice. Whereas, with SOX2OT overexpression, the above indicators were markedly decreased, suggesting a relief effect of SOX2OT on renal injury. Furthermore, renal injury, interstitial fibrosis and LC3 expression were detected by HE, Masson and immunohistochemical staining, illustrating that overexpression of SOX2OT attenuated renal pathological changes and alleviated interstitial fibrosis in DN mice (Fig. 2c). Moreover, RT-qPCR results verified that downregulation of SOX2OT was observed in the kidney of DN mice, and SOX2OT overexpressing caused a significant increase in its expression (Fig. 2d). Western blot analysis further indicated that LC3II/LC3I and Beclin1 levels in the kidney of DN mice were greatly suppressed, while p62 level was upregulated. However, these effects were reversed in response to SOX2OT overexpression (Fig. 2e). Taken together, these findings indicate a protective role for SOX2OT in DN-induced renal injury.

### SOX2OT overexpression inhibited mesangial cell proliferation and fibrosis

**Fig. 3:**
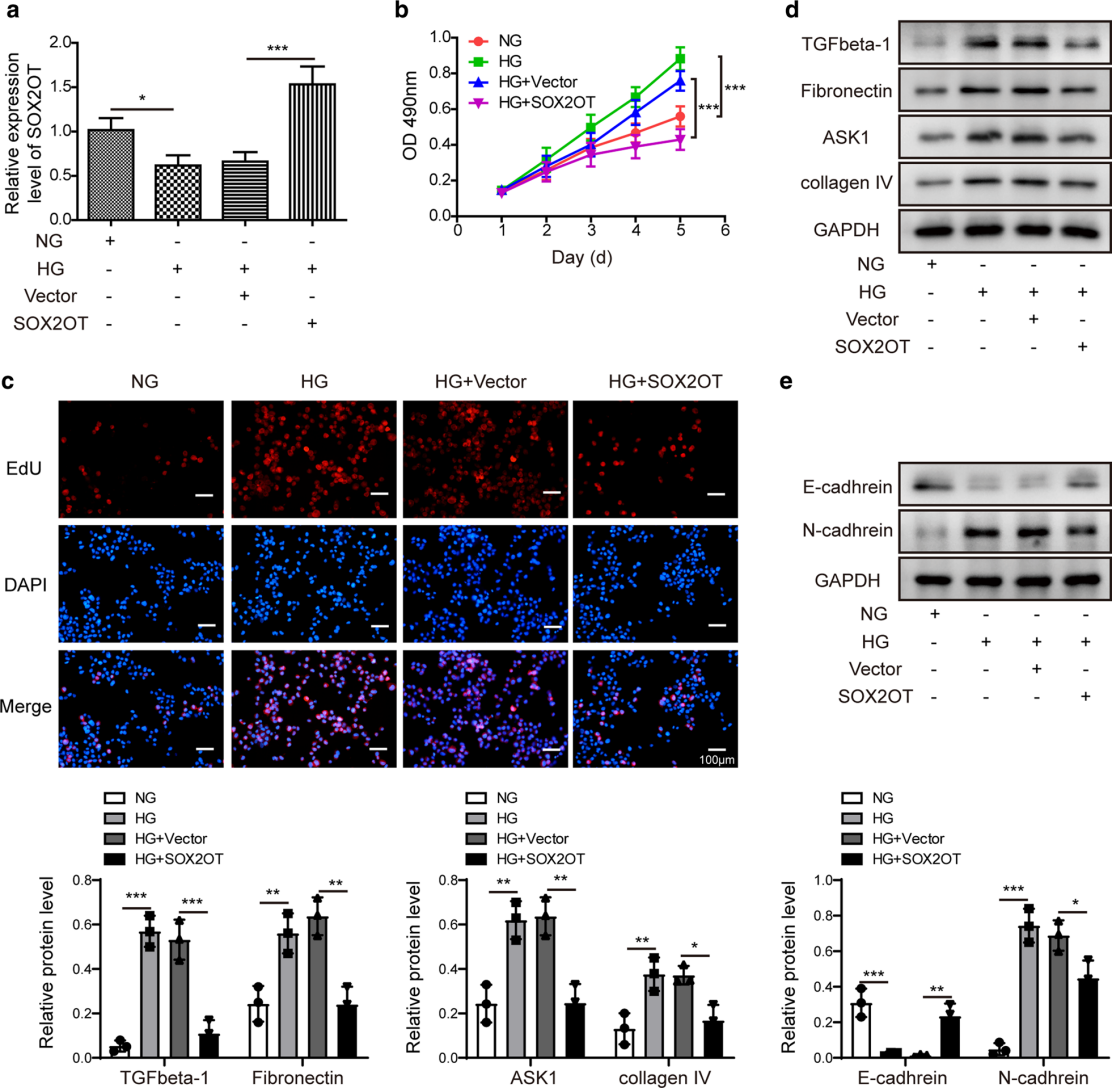
SOX2OT overexpression inhibited mesangial cell proliferation and fibrosis. Mouse mesangial cells were incubated with high glucose (HG) or normal glucose (NG). HG cells were transfected with pcDNA-SOX2OT or pcDNA-vector. **a** mRNA expression of SOX2OT was detected by RT-qPCR in HG cells after SOX2OT overexpression. **b** Cell survival was measured using CCK-8 assay after SOX2OT overexpression. **c** EDU assay was performed to assess proliferation in HG cells after SOX2OT overexpression. **d** Western blot analysis of TGFβ-1, fibronectin, ASK1 and collagen IV in HG cells after SOX2OT overexpression. **e** Western blot analysis of E-cadherin and N-cadherin in HG cells after SOX2OT overexpression. GAPDH was used as a loading control in western blot analysis. Error bars represent the mean ± SD from three independent experiments. *p < 0.05, **p < 0.01, ***p < 0.001, One-way-ANOVA followed by Newman-Keuls post hoc test

To further clarify the biological functions of SOX2OT, additional in vitro studies were performed. To explore whether SOX2OT affects mesangial cell proliferation, pcDNA-SOX2OT or vector control was transfected into cells with high glucose. RT-qPCR results indicated that SOX2OT was downregulated in HG cells, but overexpression of SOX2OT caused a significant increase in its expression (Fig. 3a). Next, CCK-8 assay implied that cell survival was enhanced in HG cells compared to NG cells, but this difference was disappeared in response to SOX2OT overexpression in HG cells (Fig. 3b). In addition, EDU assay was performed to detect proliferation of mesangial cells. EDU positive cells with high glucose were greatly increased compared to those in the NG group, while EDU positive cells in the HG group with SOX2OT overexpression were significantly reduced compared to the HG group (Fig. 3c), suggesting that SOX2OT inhibit mesangial cell proliferation. Furthermore, data from fibrosis-related indicators using western blot indicated upregulation of TGFβ-1, fibronectin, ASK1 and collagen IV in HG cells compared to NG cells, and these effects were abolished by SOX2OT overexpression (Fig. 3d). Additionally, EMT-related molecules were analysed. Results revealed that HG inhibited E-cadherin expression, but N-cadherin expression was significantly increased in HG cells. These effects were both reversed by SOX2OT overexpression (Fig. 3e). These results suggest that SOX2OT might attenuate DN-induced renal injury by regulating cell proliferation and fibrosis.

### SOX2OT promoted autophagy and relieved cell proliferation and fibrosis by inhibiting Akt/mTOR signalling

**Fig. 4:**
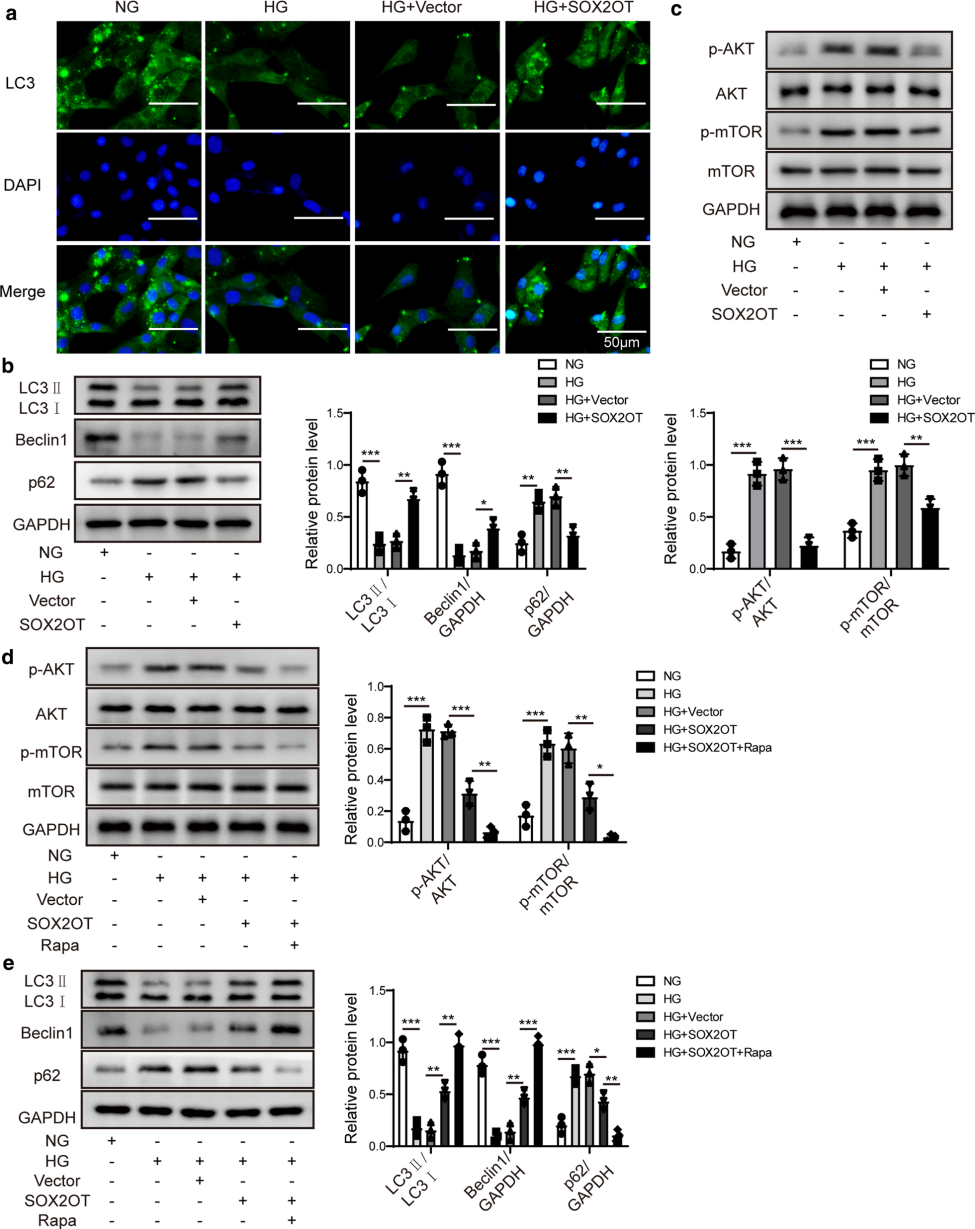

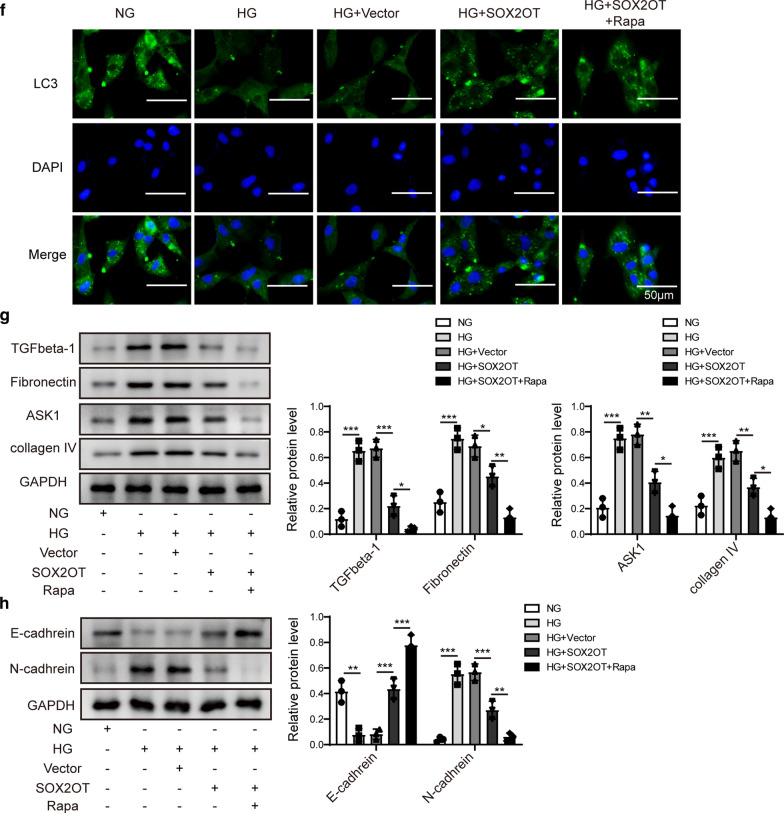
SOX2OT promoted autophagy and relieved cell proliferation and fibrosis by inhibiting the Akt/mTOR pathway. Mouse mesangial cells were incubated with high glucose (HG) or normal glucose (NG). HG cells were transfected with pcDNA-SOX2OT or pcDNA-vector. **a**, **f** LC3 expression in HG cells after SOX2OT overexpression in the presence or absence of rapamycin was assessed by immunofluorescence. Scale bar: 50 μm. **b** Western blot analysis of LC3II, LC3I, Beclin1 and p62 in HG cells with SOX2OT overexpression. **c**, **d** Expression levels of p-Akt, Akt, p-mTOR, and mTOR in HG cells with SOX2OT overexpression in the presence or absence of rapamycin were determined using western blot assay. **e** Western blot analysis of LC3II/LC3I, Beclin1 and p62 in HG cells in response to SOX2OT overexpression in the presence or absence of rapamycin. **g** Western blot assay was implemented to detect expression of TGFβ-1, fibronectin, ASK1 and collagen IV in HG cells with SOX2OT overexpression in the presence or absence of rapamycin. **h** Expression of E-cadherin and N-cadherin detected by western blot assay in HG cells after SOX2OT overexpression in the presence or absence of rapamycin. GAPDH was used as a loading control in western blot analysis. Error bars represent the mean ± SD from three independent experiments. Rapa, rapamycin. *p < 0.05, **p < 0.01, ***p < 0.001, One-way-ANOVA followed by Newman–Keuls post hoc test

We further investigated whether SOX2OT overexpression affected mesangial cell autophagy. Immunofluorescence results revealed that HG treatment reduced the fluorescence intensity of LC3, while overexpression of SOX2OT restored LC3 expression in HG cells (Fig. 4a). Moreover, expression of LC3II/LC3I and Beclin1 in HG cells was suppressed compared to NG group, while p62 was upregulated. However, these effects were reversed in response to SOX2OT overexpression (Fig. 4b), indicating that overexpression of SOX2OT promotes autophagy in HG mesangial cells.

As an essential role of Akt/mTOR signalling during cell growth and proliferation, we further investigated whether SOX2OT regulates the pathogenesis of DN through the Akt/mTOR pathway. Phosphorylation of Akt and mTOR was increased in cells with high glucose compared to the normal glucose group (Fig. 4c). However, activation of Akt/mTOR was blocked in HG cells in response to SOX2OT overexpression. To further clarify whether SOX2OT controls DN development through the Akt/mTOR pathway, rapamycin, an inhibitor of mTOR signalling, was used. Compared to the HG group with SOX2OT overexpression, Akt/mTOR activity was further inhibited after rapamycin pretreatment (Fig. 4d).

Moreover, the ratio of LC3II/LC3I and expression of Beclin1 in HG cells were significantly suppressed compared to NG cells, while p62 expression was upregulated. However, in the case of SOX2OT overexpression, these effects were reversed. These reversal effects were further enhanced after pretreatment with rapamycin (Fig. 4e), suggesting that mTOR mediates SOX2OT-induced autophagy. Consistent with western blot analysis, LC3 fluorescence intensity with both SOX2OT overexpression and rapamycin pretreatment was significantly enhanced compared to only SOX2OT overexpression in HG cells (Fig. 4f). Furthermore, protein expression of TGFβ-1, fibronectin, ASK1, and collagen IV was dramatically downregulated with both SOX2OT overexpression and rapamycin pretreatment in HG cells compared to those with only SOX2OT overexpression in HG cells (Fig. 4g). In addition, E-cadherin expression was inhibited by HG, but the N-cadherin expression was markedly increased in HG cells. However, these effects were both reversed with SOX2OT overexpression, and these reversal effects were further enhanced after pretreatment with rapamycin (Fig. 4h), suggesting that SOX2OT relieves fibrosis in HG cells through inhibiting Akt/mTOR signalling.

### SOX2OT alleviated DN pathogenesis by regulating Akt/mTOR-mediated autophagy in STZ-induced DN mice

**Fig. 5:**
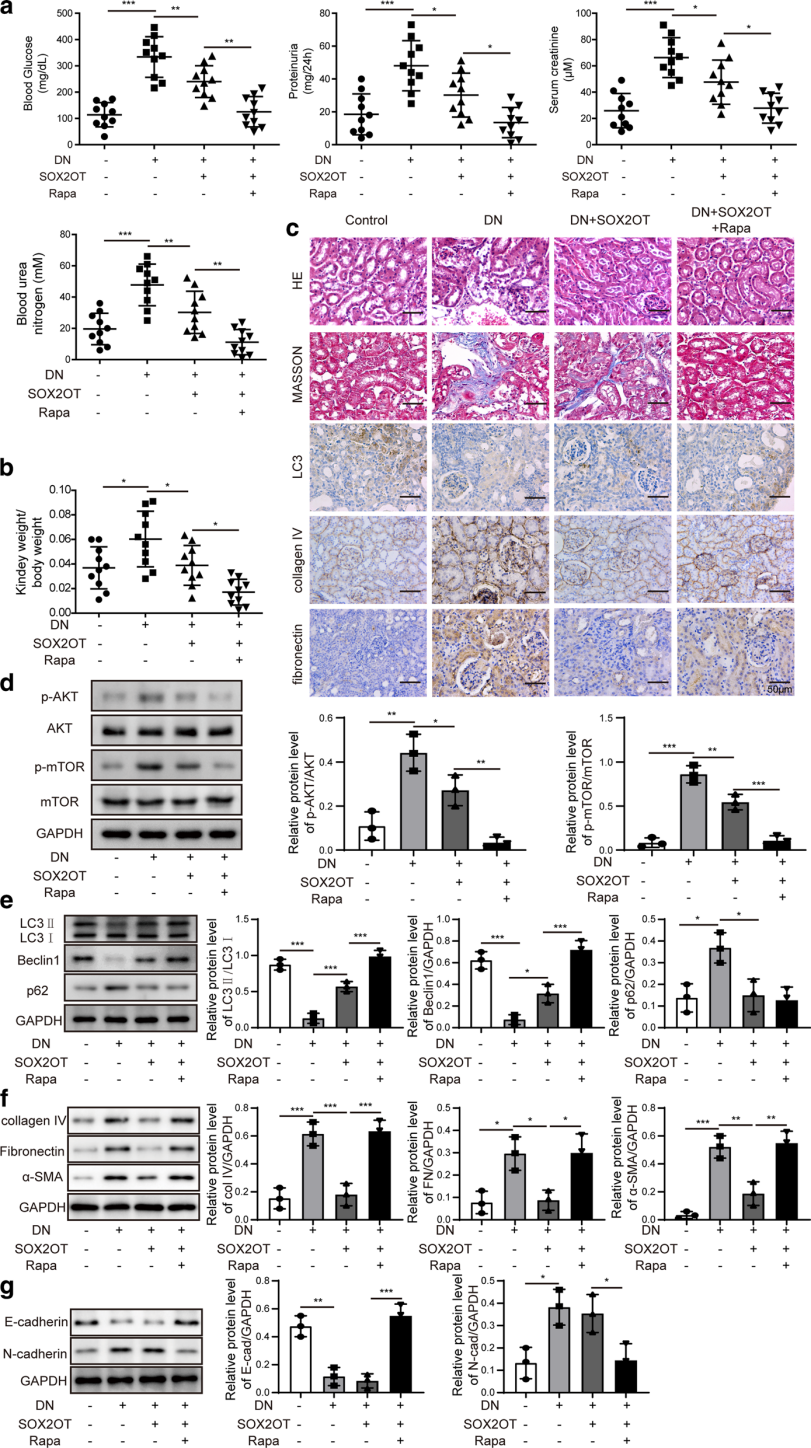
SOX2OT alleviated DN pathogenesis by regulating Akt/mTOR-mediated autophagy in STZ-induced DN mice. **a** Blood glucose, 24 h proteinuria, serum creatinine and blood urea nitrogen levels in DN mice after SOX2OT overexpression in the presence or absence of rapamycin. **b** Relative kidney weight in DN mice with SOX2OT overexpression in the presence or absence of rapamycin. **c** HE, Masson and LC3 staining of renal sections in DN mice in response to SOX2OT overexpression in the presence or absence of rapamycin. IHC staining detected fibronectin and collagen IV levels. Scale bar: 50 μm. **d** Western blot analysis was employed to determine p-Akt, Akt, p-mTOR and mTOR levels in the kidney of DN mice after SOX2OT overexpression in the presence or absence of rapamycin. **e** Western blot analysis of LC3II, LC3I, Beclin1 and p62 in the kidney of DN mice after SOX2OT overexpression in the presence or absence of rapamycin. **f**, **g** Expression levels of collagen IV, fibronectin, α-SMA, E-cadherin and N-cadherin were measured by western blot in the kidney of DN mice after SOX2OT overexpression in the presence or absence of rapamycin. GAPDH was used as a loading control in western blot analysis. Error bars indicate the mean ± SD of at least triplicate experiments. Rapa, rapamycin. *p < 0.05, **p < 0.01, ***p < 0.001, One-way-ANOVA followed by Newman-Keuls post hoc test

To verify whether SOX2OT alleviates DN pathogenesis by regulating Akt/mTOR-mediated autophagy in vivo, we treated STZ-induced DN mice with SOX2OT overexpression and rapamycin. Biochemical parameters, including blood glucose, 24 h proteinuria, serum creatinine, blood urea nitrogen and relative kidney weight were determined. Results showed that all parameters that were inhibited by SOX2OT overexpression were further decreased after treatment with rapamycin (Fig. 5a, b). Furthermore, renal morphological changes, renal interstitial fibrosis and LC3 expression were detected by HE, Masson staining and immunohistochemical staining in DN mice. Results illustrated that rapamycin treatment further enhanced the protective effects of SOX2OT overexpression on renal injury and interstitial fibrosis (Fig. 5c). In addition, IHC staining showed that SOX2OT overexpression significantly suppressed the expression of fibronectin and collagen IV which was induced in the kidney of DN mice. However, these effects were both reversed by rapamycin (Fig. 5c). Moreover, activation of Akt/mTOR signalling with both SOX2OT overexpression and rapamycin pretreatment was further inhibited compared to those with only SOX2OT overexpression in the kidney tissues of DN group (Fig. 5d). In addition, autophagy-related protein expression of LC3II/LC3I and Beclin1 in the kidney of DN group was markedly suppressed compared to the control group, while p62 levels were significantly upregulated. However, these effects were reversed by SOX2OT overexpression, and these reversal effects were further enhanced with rapamycin treatment (Fig. 5e). Additionally, SOX2OT overexpression remarkably promoted the expression of E-cadherin inhibited in DN mice, and inhibited the levels of collagen IV, fibronectin, α-SMA and N-cadherin induced in DN mice. However, these effects were both reversed by rapamycin (Fig. 5f, g). All the original images of western blot in this study have been shown in  Additional files 1 and 2. Therefore, these data indicated that SOX2OT alleviates the pathogenesis of DN via Akt/mTOR-mediated autophagy.

## Discussion

Diabetic nephropathy (DN) is a major chronic complication of diabetes, which may result in loss of renal function (Tan et al. 2019). Glomerular hypertrophy, thickening of the basement membrane and glomerulosclerosis are important pathological features of DN (Wang et al. 2018; Nagai et al. 2005). Mesangial cells are the basic components of the glomerulus, and proliferation of these cells has been identified as a primary contributing factor to renal fibrosis (Wang et al. 2018; Yang et al. 2019). Thus, inhibition of mesangial cell proliferation represents a potential target for the prevention of DN pathogenesis (Wang et al. 2016). However, the precise molecular mechanisms regulating mesangial cell proliferation are not well understood. In this study, we demonstrated that SOX2OT overexpression relieves cell proliferation and fibrosis via inhibition of Akt/mTOR signalling both in vivo and in vitro.

Autophagy plays an important role in the occurrence and development of DN (Deshpande et al. 2018; Lim et al. 2018, Liu et al. 2017). A previous study showed that proteinuria in DN was alleviated through activating podocyte autophagy (Liu et al. 2017). Moreover, another study verified that TGF-β and miR-192 reduced autophagy in kidney glomerular mesangial cells under diabetic conditions (Deshpande et al. 2018). In accordance with these previous studies, we found that overexpression of SOX2OT enhanced mesangial cell autophagy and relieved DN-induced renal injury.

Many studies have demonstrated the growing importance of lncRNAs in DN development and renal fibrosis prevention (Long and Danesh 2018; Hu et al. 2017; Liu et al. 2019; Wang et al. 2019). For example, p53 could attenuate renal dysfunction in DN via inhibiting lncRNA ZEB1-AS1 (Wang et al. 2018). Wang et al. reported that lncRNA CYP4B1-PS1-001 inhibited the proliferation and fibrosis of mouse mesangial cells through regulating the ubiquitination and degradation of nucleolin in DN (Wang et al. 2018). LncRNA SOX2OT is involved in various biological processes, such as proliferation, differentiation and metastasis (Shahryari et al. 2015). Abnormal expression of SOX2OT has been observed in various of cancers (Han et al. 2018; Zhang et al. 2016). Results of microarray analysis indicated that expression of SOX2OT is decreased in DN (Zhang et al. 2018). Similarly, our data suggest that SOX2OT expression is downregulated in DN mice and high glucose-induced mouse mesangial cells. In addition, SOX2OT overexpression markedly repressed DN-induced renal injury. Meanwhile, we observed that overexpression of SOX2OT remarkably suppressed proliferation and fibrosis in mesangial cells, suggesting a protective function for SOX2OT on DN. Convincing evidence suggests that SOX2OT plays a vital role in cell proliferation, apoptosis and regeneration in diabetes related diseases in vitro and in vivo. For instance, in diabetic retinopathy, retinal ganglion cells undergo cell apoptosis due to high glucose stress. SOX2OT knockdown could increase retinal ganglion cell viability, and decrease high glucose induced retinal ganglion cell apoptosis. Moreover, SOX2OT knockdown could play a neuroprotective role in diabetic mouse model (Li et al. 2017). On the other hand, one study from Zhang et al. showed a protective role of SOX2OT in high glucose-induced podocytes injury in DN. They found that SOX2OT overexpression alleviated the high glucose-induced podocytes injury through autophagy induction by the miR-9/SIRT1 axis (Zhang et al. 2019)**.** In this study, we also verified the alleviating effect of SOX2OT on the proliferation and fibrosis of glomerular mesangial cells induced by high glucose. However, one of the limitations in this study was that we haven't precisely pinpointed within the animal level in which cell type SOX2OT specifically exerted its critical biological functions. Further research should be performed in vivo to clarify the main cell type in which SOX2OT ultimately plays a role.

We further evaluated the underlying mechanism for these observations in detail. Previous studies have reported that mTOR acts as a negative regulator of autophagy (Martina et al. 2012, Fantus et al. 2016). Rapamycin, an inhibitor of mTOR, has been reported to prevent the early steps of DN (Chen et al. 2005; Lieberthal and Levine 2009; Mori et al. 2009; Yang et al. 2007). For example, rapamycin reduces the morphological and functional disorders in diabetic kidneys (Chen et al. 2005). In addition, Akt/mTOR is reportedly highly activated in DN (Nagai et al. 2005; Mavroeidi et al. 2013). For instance, phosphorylated/activated forms of AKT (Thr308) and mTOR (Ser2448) were suppressed by losartan in type 1 diabetic nephropathy (Mavroeidi et al. 2013). Similarly, our data demonstrated that phosphorylation of Akt and mTOR was induced in HG mesangial cells. However, overexpression of SOX2OT significantly inhibited Akt and mTOR activity. Elevation of LC3II by SOX2OT overexpression was enhanced by incubation with rapamycin, suggesting that mTOR mediates SOX2OT-induced autophagy. In addition, we have observed that SOX2OT reduced blood glucose in DN mice. Diabetes and its secondary pathologies are a series of pathological processes caused by changes in the body's internal environment. Blood glucose is used as a test indicator to judge the severity of the condition (Ehrhardt and Al Zaghal 2019) and lncRNAs play an important role in the regulation of glucose homeostasis and diabetes (Sun and Wong 2016). Based on the data from the present study, we speculate that it may be due to the therapeutic effect of SOX2OT overexpression on kidney tissues, which improved the internal environment of the body and finally caused the alteration of detection indicators such as blood glucose, 24-h proteinuria, serum creatinine, and urea nitrogen. For the specific mechanism of SOX2OT protecting kidney, we set out to explore it from the perspective of autophagy in this study. Autophagy is a highly conserved cellular process that transports macromolecules and other damaged organelles to the lysosome for degradation and recycling to maintain intracellular homeostasis. Studies have confirmed that autophagy plays a critical role in maintaining intracellular lysosomal homeostasis under diabetic conditions (Lim et al. 2018). Indeed, the shortcoming of this study is that it did not reveal the specific target of SOX2OT in regulating Akt/mTOR signal. It has been shown that lncRNAs can act as the sponges of microRNAs in mesangial cells to regulate gene expression, hence, affect DN (Ge et al. 2019; Ji et al. 2019). Further research should be performed to explore the direct downstream targets of SOX2OT in mesangial cells, such as microRNAs, that translate into cellular functional effects.

## Conclusions

In conclusion, our study demonstrated that lncRNA SOX2OT was downregulated in both animal and cell models of DN. SOX2OT overexpression alleviated DN via autophagy induction by inhibition of the Akt/mTOR pathway. These findings provide a better understanding of the mechanism of SOX2OT in DN and strongly indicate that SOX2OT represents a therapeutic target for DN. However, future research is needed to identify the specific signalling pathways mediated by SOX2OT/Akt/mTOR. Besides, given the diversity of regulatory mechanisms and molecular targets of lncRNAs, whether this pathway is unique to mesangial cells or if SOX2OT influences the development of DN through the miR-9/SIRT1 axis have not been identified yet.

## Supplementary Information

**Additional file 1.** The original images of western blot designed in the study.

**Additional file 2.** The original images of western blot designed in the study.

## Data Availability

All data generated or analyzed during this study are included in this published article (Additional files 1 and 2).

## Abbreviations

DN: Diabetic nephropathy
IHC: Immunohistochemistry
lncRNAs: Long non-coding RNAs
SOX2OT: LncRNA SOX2 overlapping transcript
STZ: Streptozotocin
SPF: Specific-pathogen-free
CCK-8 assay: Cell Counting Kit-8 assay
EDU assay: 5-Ethynyl-2′-deoxyuridine assay
