# Supplementary figures and images for: LncRNA SOX2OT alleviates mesangial cell proliferation and fibrosis in diabetic nephropathy via Akt/mTOR-mediated autophagy
Source: Mol Med. 2021 Jul 8;27:71. doi: 10.1186/s10020-021-00310-6 (PMC8268185; doi:10.1186/s10020-021-00310-6)

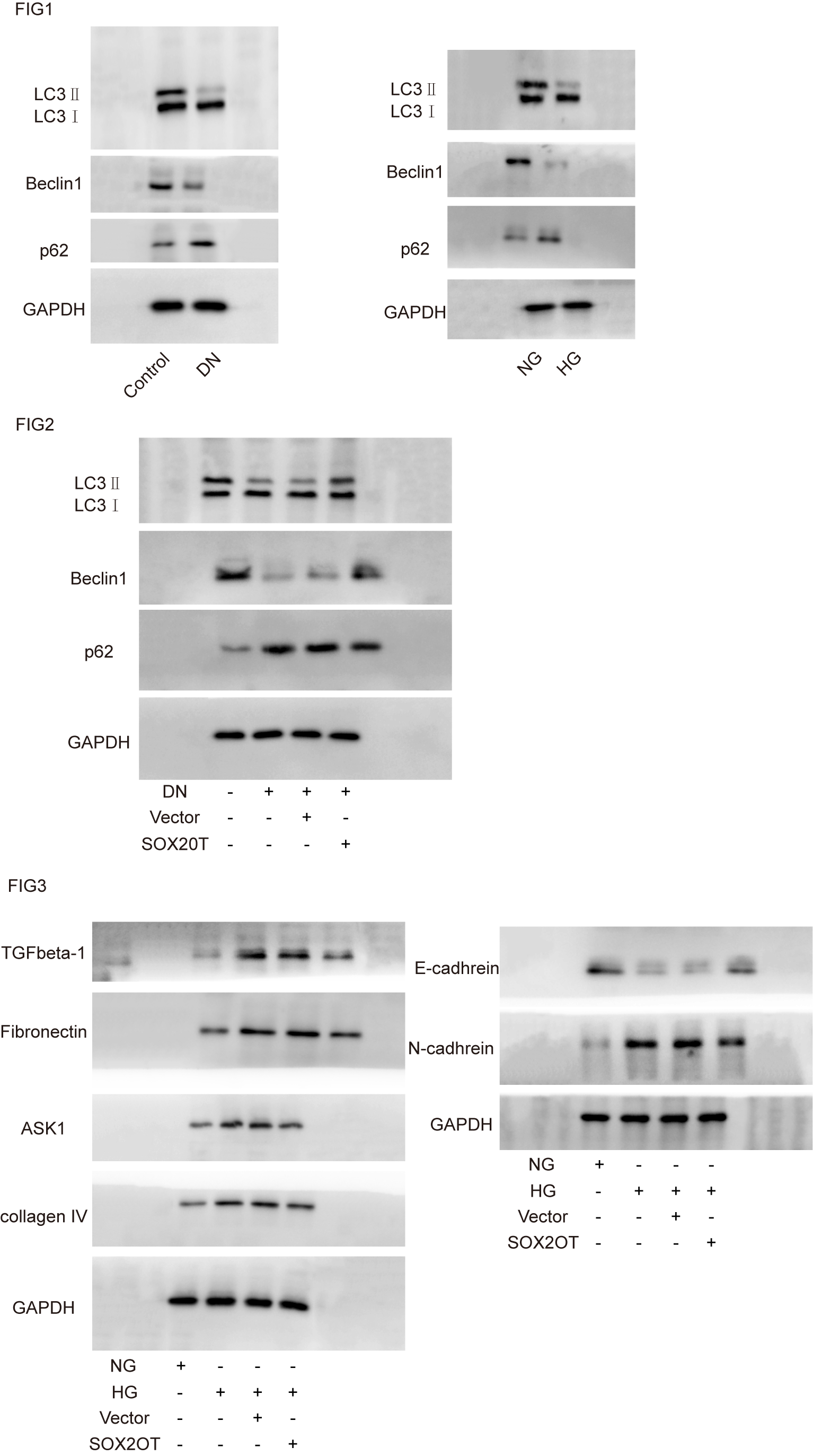

Supplement: Supplementary file 1 — Additional file 1. The original images of western blot designed in the study. [file 10020_2021_310_MOESM1_ESM.tif]

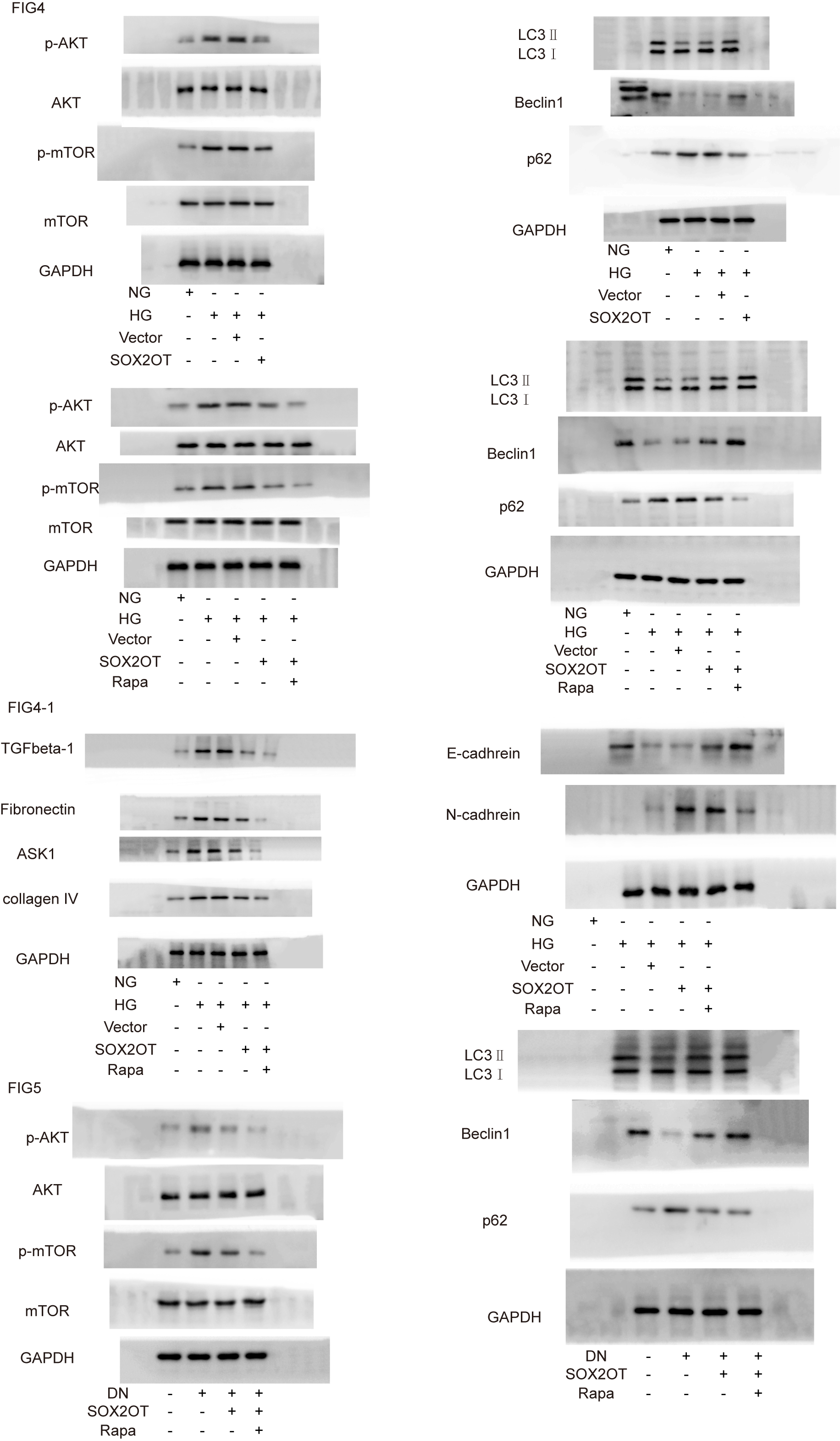

Supplement: Supplementary file 2 — Additional file 2. The original images of western blot designed in the study. [file 10020_2021_310_MOESM2_ESM.tif]
